# Comparing the quality of video and phone consultations in primary care telemedicine: a cross-sectional study of over 180,000 appointments

**DOI:** 10.1186/s12875-026-03454-8

**Published:** 2026-07-13

**Authors:** Daniel Beirão, Filipe Freitas Pinto, João Magalhães, Carlos Franclim Silva, Bernardo Sousa-Pinto, Ana Luísa Neves, Paulo Santos

**Affiliations:** 1 MEDCIDS, Faculty of Medicine, University of Porto, Alameda Prof. Hernâni Monteiro, Porto, 4200-319 Portugal; 2 RISE-Health, Faculty of Medicine, University of Porto, Alameda Prof. Hernâni Monteiro, 4200-319 Porto, Portugal; 3 knok Healthcare, Rua Mouzinho de Albuquerque 742, Matosinhos, Porto, 4450-007 Portugal; 4 Department of Primary Care and Public Health, Imperial College London, London, UK

**Keywords:** Telemedicine, Primary health care, Quality of health care, Patient reported outcomes

## Abstract

**Background:**

Telemedicine has gained prominence in primary care, but evidence comparing its modalities is scarce.

**Methods:**

We conducted a cross-sectional study of 184,308 video and phone consultations from a telemedicine platform in Portugal, comparing quality indicators based on the Institute of Medicine (IOM) domains.

**Results:**

Video consultations were associated with lower recurrence rates, higher patient-reported helpfulness (95.4% vs. 92.3%), and greater satisfaction. Time to appointment was shorter for phone consultations, but video had a longer duration.

**Conclusions:**

In this cross-sectional analysis, video consultations were associated with higher patient-reported helpfulness and lower short-term recurrence than telephone consultations, while telephone offered faster access. Because telephone users were substantially older and the dataset lacked measures of comorbidity, illness severity, and socioeconomic status, these associations are likely confounded by indication and should not be read as causal evidence of superior quality. The findings nonetheless point to differences between modalities and to the importance of equitable access to information-richer consultations.

**Supplementary Information:**

The online version contains supplementary material available at https://doi.org/10.1186/s12875-026-03454-8.

## Background

Telemedicine appointments have become relevant to healthcare delivery in recent years, particularly in primary care settings [1, 2]. The rise of telemedicine can be attributed to digital technology advances, increased internet access, and a growing need for convenient and accessible healthcare options [3]. Telemedicine offers important benefits, including reduced travel time and patient costs, increased access to care for remote areas, and improved flexibility for healthcare providers [4–6]. Telemedicine is crucial to ensuring the sustainability of healthcare systems, particularly in contexts of high demand and low resource availability [7].

Telemedicine appointments can be conducted through various modalities, with video and phone consultations being the most common methods. These two approaches have distinct features, which can lead to differences in the quality and effectiveness of healthcare delivery. Video consultations may provide a more interactive experience and could support more comprehensive patient assessment [8]. Through real-time video communication, providers can observe patients’ physical appearance, body language, and other non-verbal cues, which may contribute to more effective doctor-patient communication [2, 9, 10]. Additionally, video consultations enable healthcare providers to demonstrate physical examinations or procedures and allow patients to visually share any relevant information, such as wounds or rashes [11]. On the other hand, phone consultations are associated with more convenience and ease of access. However, the lack of visual information may reduce the scope of assessment and diagnostic accuracy, leading to suboptimal treatment plans. Both modalities may also be less suitable for patients with hearing impairment or language barriers, posing challenges to communication and understanding.

Despite the well-recognized benefits of telemedicine, some challenges often lead to failure during the implementation phase of new telemedicine initiatives [1]. Video and phone telemedicine consultations have been insufficiently compared, particularly regarding their quality, patient outcomes, and satisfaction [1]. Most available studies on telemedicine focused on comparing both video or phone consultations with face-to-face consultations [5, 6, 12–17]. The available studies comparing phone and video consultations mainly focused on patient sociodemographic characteristics [18, 19], had relatively small sample sizes [18], and samples poorly representative of the overall population [16]. Finally, some of these studies relied on data collected exclusively during the COVID-19 pandemic [18–21], which may be strongly biased by a specific clinical, epidemiological, organizational, and social context.

In this study, we aimed to compare video and telephone consultations on quality, clinical outcomes, and patient satisfaction in the context of primary care telemedicine. In addition, we mapped the assessed variables in the quality domains (effectiveness, patient-centeredness, timeliness, efficiency, and equity) outlined as essential for the improvement of healthcare systems by the Institute of Medicine (IOM, currently known as the National Academy of Medicine, https://nam.edu/).

## Methods

### Overview

This cross-sectional study compared video with phone telemedicine appointments in primary care settings. Phone telemedicine appointments were defined as those synchronous medical consultations performed by call (via telephone, mobile phone, or any suitable device), with voice but not video transmission. Video telemedicine appointments differed from phone appointments by adding synchronous image (video) transmission. Both communication channels were provided through knok’s telemedicine platform. The patient experience was the same for both services (phone and video consultation), in what refers to the booking process; the booking alerts via SMS and e-mails to avoid no-shows and guide patients in the platform use; the digitalized prescriptions of medication and exams; two-way protected file sharing; experience assessment.

We focused on quality, clinical outcomes, and patient satisfaction (primary outcomes). Additionally, we addressed the patients’ socio-demographic distribution (secondary outcome) in each type of telemedicine provision (phone and video appointments).

### Setting and participants

We assessed data from telemedicine consultations provided through knok’s platform. knok is a private, digital-first telemedicine provider that delivers synchronous primary care consultations predominantly through private health-insurance and corporate-health pathways, operating alongside — rather than within — the public National Health Service (Serviço Nacional de Saúde). knok operates nationally rather than within a single region, serving a predominantly privately insured population; in 2023, private voluntary health insurance in Portugal covered approximately 3.8 million people, around a third of the population [22]. This affords the cohort broad national reach beyond the major urban centres, although privately insured and digitally engaged patients are over-represented relative to the general population, as we address in the Limitations. Patients book consultations through the platform and select the consultation modality, video (synchronous audiovisual) or telephone (audio-only), at the time of booking; the platform presents both options and no clinical triage or algorithmic rule assigned the modality. The platform provides integrated digital prescribing of medicines and diagnostic tests, two-way secure file sharing, and automated booking reminders, with an identical booking experience across modalities.

We collected data from all completed primary care appointments (including video and phone consultations) from Portugal-based patients from March 2021 through March 2023. When a patient required a legal tutor due to (i) pre-existing impairments (e.g., major cognitive deficits) or (ii) age below 16 years, the consultation was conducted in the presence of the legal tutor, according to the terms of service use. The analysis did not consider data from patients who did not attend the booked telemedicine appointment (no-shows). Consultations with a recorded duration of one minute or less were excluded as incomplete or non-genuine encounters (for example, dropped connections or test bookings); all included consultations had an internal platform status of “finished”.

### Data sources

Our primary data source was electronic health records (EHRs) from knok´s platform. All data collection procedures complied with applicable privacy and security regulations. Following each telemedicine appointment, patient satisfaction and clinician opinion were assessed using a standardized questionnaire presented in the telemedicine platform and sent automatically by email at the end of the consultation.

Patients received a review questionnaire asking whether the telemedicine appointment was helpful or not (patient-reported consultation helpfulness), with a binary yes/no response. Moreover, patients were asked to rate the probability of recommending knok to someone (patient-reported appointment classification) on a scale from 1 (lowest satisfaction; not at all likely to recommend the service to others) to 10 (highest satisfaction; extremely likely to recommend the service to others), used to determine Net Promoter Score (NPS) [23].

Doctors received a questionnaire asking if they considered that the patient needed an in-person appointment to provide assistance for the same reason of the teleconsultation (excluding the need for complementary tests or follow-up appointments).

These post-consultation prompts were brief, single-item items designed for routine quality monitoring, without multi-item scales or psychometric validation; the exact English wording is provided in Supplementary File S1 (Questionnaire — English translation). Data were collected and de-identified before analysis to ensure patient privacy.

### Variables

The variables of interest for our study included socio-demographic data of the patient (age and sex), time to appointment (minutes), appointment duration (minutes), patient-reported consultation helpfulness, patient-reported appointment classification; and clinical variables such as recurrence, defined as any new appointment request by the same patient to the knok platform within 24, 48, or 72 h or seven days of the index consultation, identified through unique patient identifiers, and doctor-reported need for an in-person evaluation (after consultation). Recurrence captured intra-platform contacts only and was operationalised independently of the diagnostic code of the subsequent contact.

Variables of interest were mapped across the quality domains defined by the IOM (effectiveness, patient-centeredness, timeliness, efficiency, and equity), as presented in Table 1.

**Table 1:** Description of study variables and mapping according to IOM domains of healthcare and the rationale for each domain assignment

Variable	Description
Equity: Providing care that does not vary in quality because of personal characteristics such as gender, ethnicity, geographic location, and socio-economic status. *Rationale*: Age and sex are examined not as quality outcomes in themselves but to operationalise equitable access — to detect whether the modality offering richer clinical information (video) was distributed unequally across demographic groups, a digital adaptation of the inverse care law [31].	
Age	Age at time of the appointment
Sex	Biological sex
Timeliness: Reducing waits and sometimes harmful delays for both those who receive and those who give care. *Rationale*: Time to appointment indexes the promptness of care delivery and the system’s capacity to mitigate logistical delay, identified as one of the principal patient-valued benefits of remote consultation [16].	
Time to appointment	Time from first (administrative) contact to the beginning of the consultation with a doctor
Efficiency: Avoiding waste, including waste of equipment, supplies, ideas, and energy. *Rationale*: Consultation duration and short-term recurrence index resource use [30]; recurrence additionally serves as a pragmatic proxy for first-contact resolution, where short-term re-access may represent failure demand — the re-work generated when a first contact does not resolve the problem — but may also reflect value demand, such as planned follow-up (see Limitations).	
Duration	Time between the beginning and the end of a consultation with a doctor
Recurrence	New consultation in the next 24, 48, or 72 h or in the next 7 days
Patient-centeredne**ss**: Providing care that is respectful of and responsive to individual patient preferences, needs, and values and ensuring that patient values guide all clinical decisions. *Rationale*: Appointment helpfulness and the recommendation rating capture the patient’s own perspective on the encounter, the construct central to this domain; both are single-item, non-validated prompts used for routine quality monitoring (see Methods) [4].	
Appointment helpfulness	Described by patients as being helpful (patients’ perspective)
Appointment classification	Described by patients as a reflection of satisfaction
Effectiveness: Providing services based on scientific knowledge to all who could benefit and refraining from providing services to those not likely to benefit (avoiding underuse and overuse, respectively). *Rationale*: The doctor-reported need for subsequent in-person evaluation indexes the resolution capacity of the remote contact — whether the encounter was clinically sufficient or required subsequent in-person assessment [6]. In this cohort the indicator was higher for video, interpreted as greater case-mix complexity rather than lower effectiveness (see Discussion).	
Need for in-person evaluation	Doctor-reported need for patients observed in a telemedicine appointment being oriented to in-person care

Domain definitions are reproduced from the IOM framework [24]

*IOM* Institute of Medicine (now the National Academy of Medicine)

This mapping operationalises the subset of IOM domains measurable in routinely collected platform data and is a pragmatic operationalisation rather than a validated instrument [24]. Age and sex were placed under Equity not as quality outcomes in themselves, but to operationalise equitable access — that is, to detect whether the modality offering richer clinical information was distributed unequally across demographic groups (a digital adaptation of the inverse care law). Time to appointment was mapped to Timeliness, focusing on the promptness of healthcare delivery. Duration and recurrence at 24 h, 48 h, 72 h, and seven days were linked to Efficiency because they index resource use and, for recurrence, failure demand — the re-work generated when a first contact does not resolve the problem. Appointment helpfulness and classification fell under Patient-centeredness, highlighting the need for care that is respectful of and responsive to individual patient preferences, needs, and values. Finally, the need for an in-person appointment was associated with Effectiveness, ensuring that healthcare services were based on scientific knowledge and resulted in improved health outcomes.

### Statistical analysis

Categorical variables were described using absolute and relative frequencies, and continuous variables were described using medians and interquartile ranges (IQR). To assess differences in patient characteristics between video and phone appointments, we built mixed-effects regression models for each of the outcome variables, having the type of telemedicine consultation as an independent variable and the identification of the patient as a random effect (that is, we clustered those observations belonging to the same patients). For continuous or ordinal outcome variables (time to appointment, appointment duration, and appointment classification by patients), we built mixed-effects linear regression models. For categorical outcome variables (whether the appointment was helpful, recurrence, and the need for in-person evaluation), we built mixed-effects logistic regression models. We built both univariable and multivariable regression models, adjusting for patients’ age, gender, and semester. Demographic factors such as age [25] and gender [26] may influence telehealth usage, so these variables were included as covariates, and semester was added to account for seasonality in telehealth services. Logistic regression coefficients were interpreted as OR.

Statistical analyses were performed using R [27], version 4.2.2. A p-value of less than 0.05 was considered statistically significant. We did not perform database corrections, such as imputation techniques, to deal with missing data. Missing data were, therefore, removed from the database.

### Data reporting

This study followed the guidance on reporting standards from the Strengthening the Reporting of Observational Studies in Epidemiology (STROBE) guidelines [28].

## Results

During the studied period, there were a total of 184,308 teleconsultations in primary care settings, of which 101,941 (55.3%) were video consultations and 82,367 (44.7%) were phone consultations. These consultations were contributed by 100,117 unique patients, 34.4% of whom had more than one consultation during the study.

The descriptive analysis of these consultation variables is presented in Table 2. Most consultations concerned female participants in video (53.8%) and phone (66.0%) modalities. Patients’ median age was 37 years (IQR = 30), lower in the video consultations group (median = 31 years; IQR = 23) than in the phone consultations group (median = 48 years; IQR = 37).

**Table 2:** Descriptive analysis of variables and results of univariable and multivariable models comparing video versus phone consultations

Variable	Number of valid observations	Value	Video consultations	Phone consultations	Univariable regression model		Multivariable regression model ^a^	
	*N* = 184,308		*N* = 101,941	*N* = 82,367	Coefficient(95% CI) [*p*-value]	Odds ratio(95% CI) [*p*-value]	Coefficient(95% CI) [*p*-value]	Odds ratio(95% CI) [*p*-value]
Sex – n (%)	184,308	F	54,855 (53.81%)	54,343 (65.98%)	-	-	-	-
Age – median (IQR)	182,653	years	31 (23)	48 (37)	-	-	-	-
Time to appointment – median (IQR)	184,263	minutes	84.6 (113)	61.6 (85.7)	75.0 (70.1; 79.9) [**<0.001**]	-	65.6 (60.6; 70.6) [**<0.001**]	-
Duration – median (IQR)	184,308	minutes	8.28 (6.35)	5.97 (5.12)	1.53 (0.04; 3.01) [**0.044**]	-	1.13 (-13.51; 15.77) [0.131]	-
Appointment Helpfulness – *n* (%)	42,417	Yes	29,970 (95.4%)	10,161 (92.3%)	-	2.915 (2.912; 2.918) [**<0.001**]	-	3.16 (2.16; 4.60) [**<0.001**]
Appointment Classification – median (IQR)	43,754	1–10	10 (0)	10 (0)	0.26 (0.22; 0.29) [**<0.001**]	-	0.23 (0.22; 0.23) [**<0.001**]	-
Recurrence 24 h – *n* (%)	184,308	Yes	4308 (4.2%)	6109 (7.4%)	-	0.62 (0.60; 0.64) [**<0.001**]	-	0.62 (0.59; 0.65) [**<0.001**]
Recurrence 48 h – *n* (%)	184,308	Yes	6295 (6.2%)	8315 (10.1%)	-	0.66 (0.64; 0.68) [**<0.001**]	-	0.64 (0.62 ;0.67) [**<0.001**]
Recurrence 72 h – *n* (%)	184,308	Yes	7718 (7.6%)	9836 (11.9%)	-	0.68 (0.66; 0.71) [**<0.001**]	-	0.67 (0.64; 0.69) [**<0.001**]
Recurrence 7 days – *n* (%)	184,308	Yes	10,926 (10.8%)	13,782 (16.7%)	-	0.69 (0.67; 0.71) [**<0.001**]	-	0.66 (0.64; 0.68) [**<0.001**]
Need for in-person evaluation – *n* (%)	77,892	Yes	5041 (14.4%)	5358 (12.5%)	-	1.116 (1.059;1.1762) [**<0.001**]	-	1.116 (1.115;1.117) [**<0.001**]

Missing data for the variable Sex: 11,348 (11.13%) for video; 916 (1.11%) for phone consultations

*CI* Confidence interval, *F* Female, *IQR* Interquartile range

^a^ Regression model adjusted for patients’ age, gender, and semester

Bold values denote statistical significance (*p* < 0.05)

### Outcomes of consultations

Time to the appointment was shorter for phone consultations (median 61.6 min; IQR = 85.7) compared to video consultations (median 84.6 min; IQR = 113), with this difference being significant after adjusting for patients’ age, gender, and consultation semester (regression coefficient = 65.6; 95%CI = 60.6;70.6; *p* < 0.001). On the other hand, video consultations tended to be longer (median 8.28 min; IQR = 6.35) than phone consultations (median 5.97 min; IQR = 5.12), but multivariable-adjusted model results did not reach significance (regression coefficient = 1.1; 95%CI=-13.5;15.8; *p* = 0.131).

The vast majority of consultations were perceived as helpful by patients, with a higher approval rate for video consultations (95.4%) compared to phone consultations (92.3%) (adjusted OR = 3.2; 95%CI = 2.2;4.6; *p* < 0.001).

As indicated by the appointment classification, patient satisfaction was equally high for both modalities, with a median score of 10 out of 10. There were, however, more favorable results for video consultation in adjusted multivariable models (regression coefficient = 0.23; 95%CI = 0.22;0.23; *p* < 0.001).

Recurrence rates within 24 h of consultation were lower for video (4.2%) compared to phone consultations (7.4%) (adjusted OR = 0.62; 95%CI = 0.59;0.65; *p* < 0.001), a trend that continued for 48-hour (6.2% vs. 10.1%; adjusted OR = 0.64; 95%CI = 0.62;0.67; *p* < 0.001), 72-hour (7.6% vs. 11.9%; adjusted OR = 0.67; 95%CI = 0.64;0.69; *p* < 0.001), and 7-day recurrence rates (10.8% vs. 16.7%%; adjusted OR = 0.66; 95%CI = 0.64;0.68; *p* < 0.001).

The doctor-reported need for an in-person evaluation was slightly higher for video consultations (14.4%) compared to phone consultations (12.5%) (adjusted OR = 1.116;95%CI = 1.115;1.117; *p <* 0.001).

## Discussion

To the best of our knowledge, this is one of the largest studies to evaluate the quality of telemedicine (video and phone) appointments in primary care settings. Using a dataset of over 180,000 consultations, our study provides robust insights into the current telemedicine landscape.

Our results might be interpreted in light of the quality domains defined by the IOM (effectiveness, patient-centeredness, timeliness, efficiency, and equity) [10]. In our study, only 13.4% of patients needed an in-person evaluation after video or phone consultations, suggesting the effectiveness of these approaches. Most patients satisfied their needs during the appointment, meaning the possibility of understanding and managing the patients’ complaints during the contact. Telemedicine must be founded on evidence-based practices consistent with established clinical guidelines. We expected that using video calls would offer greater effectiveness in many cases, while phone consultations might be sufficient for routine follow-up or simple consultations. Interestingly, phone consultations were better assessed than video consultations in the effectiveness dimension when considering the doctor-reported need for in-person appointments after teleconsultation. One plausible explanation is that case mix differed between modalities, with phone consultations potentially involving a higher proportion of lower-complexity contacts (e.g., prescription renewals or test requests), though this could not be examined directly in the present analysis.

Telemedicine should be responsive to individual patient needs, preferences, and values and foster a solid patient-provider relationship. Both video and phone consultations should prioritize clear communication, shared decision-making, and emotional support. In this study, we evaluated the patient-centeredness of both phone and video modalities through the perspective of patients (appointment helpfulness and appointment classification). We observed that patient satisfaction was very high for both consultation modalities, with an estimated classification for patients reporting satisfaction scores higher in video consultations. We also found that patients deemed video consultations more helpful, possibly because the visual element allows for better patient assessment and interpretation of non-verbal cues and makes it easier for doctors to demonstrate specific procedures or concepts to patients. This is in line with Guzman et al. [9] suggestion that the visual element, such as non-verbal communication, can improve the relationship with the patient. Moreover, a study by Bleyel et al. [29] focused on mental health specialized video consultations in primary care and concluded that training medical doctors in consistent response to non-verbal cues can ensure optimal similarity to the face-to-face consultation mode.

Telemedicine appointments should be accessible and timely, reducing waiting times and providing prompt care. Both video and phone consultations can improve timeliness by eliminating travel and reducing logistical barriers, potentially leading to faster diagnosis and treatment. In an exploratory study comparing the quality of video, phone, and face-to-face consultations in primary care in the United Kingdom, patients identified shorter time to appointment as one of the main benefits of video and phone consultations [16]. This substantiates the relevance of timeliness being included as a quality domain in telehealth. This study’s results show that the time to appointment was shorter for phone consultations than for video consultations. In any case, patients could have a consultation less than 2 h after the initial contact, which is a very good indicator, as supported by the high patient satisfaction results.

Another goal telemedicine should target is optimizing resource utilization and minimizing waste. It is well-established that video and phone consultations can improve efficiency [30] by saving time, reducing the need for physical space, lowering overhead costs, and allowing healthcare providers to see more patients in the same amount of time. Even though in our study the duration of video consultations was longer than that of phone consultations, the adjusted model revealed no significant differences. Recurrence rates, in turn, were significantly lower in video consultations at all assessed time points; within the efficiency domain this is consistent with greater efficiency of the video modality, although this association is likely confounded by the older age and greater clinical complexity of telephone users and should not be interpreted causally (see Limitations).

Telemedicine should also provide care that does not vary in quality based on personal characteristics or socioeconomic factors. Both video and phone consultations can help promote equity and bridge the gap in access to care for underserved populations, such as those in rural areas or with limited mobility. However, it is essential to address potential barriers, such as digital literacy and access to technology, to ensure equitable access to telemedicine services [10, 31]. In this study, we observed that older patients tended to choose phone consultations over video consultations. This is probably explained by the still-existing grey digital divide, where younger individuals are more comfortable using new digital tools. During the study period, digital connectivity in Portugal was high but not universal: in 2022, approximately 88% of households had home internet access, 84.6% a broadband connection, and 81.8% of individuals aged 16–74 used a mobile phone or smartphone to access the internet (Statistics Portugal). [32] Because knok serves a predominantly privately insured, digital-first population, its users are likely more connected than the general Portuguese population, which both limits the generalisability of these findings and means residual confounding by digital access cannot be excluded. We suggest that as digital literacy spreads across age groups, the preference for video consultations may continue to grow. Other studies have observed similar trends [18, 19]. Of note, the age factor seems to have a similar impact on primary care providers, as shown in a cross-sectional study conducted in 16 European countries, where the adoption of video consultation was inversely associated with the seniority of the primary care provider [33]. Our analysis also revealed differences in the gender distribution of video and phone consultations, showing that women are more likely to use telemedicine services but with a slightly higher preference for the phone modality than men. There are several studies comparing phone or video consultations with face-to-face visits. The systematic review of Albornoz et al. [6] found that phone and video consultations were equivalent to face-to-face visits in improving clinical outcomes for adults with mental health conditions and those accessing primary care services. Despite a generally high level of satisfaction reported for both modalities, the review reveals significant patient dropout rates, underscoring the notion that telemedicine may be more appropriate for specific patient groups. The study highlights the critical role of a proper implementation strategy, advocating measures such as training healthcare professionals and skillful management of technical issues to ensure optimal clinical interventions.

Hammersley et al. [16] study of video, phone, and face-to-face consultations in Scottish primary care reveals a nuanced dynamic. While video and phone consultations were shorter and addressed fewer issues than their face-to-face counterparts, they were particularly attractive to individuals with work commitments, mobility, or mental health challenges. This suggests potential benefits for specific patient populations. The study draws attention to the urgent need for careful implementation and evaluation of video consultations in primary care to reap their full benefits. Aligned with this concept, Jimenez-Rodriguez [34] formulated guidelines for healthcare professionals to effectively handle video consultations across various stages: preparation, consultation, and post-consultation. These criteria garnered consensus among the participating health professionals.

These perspectives underscore the importance of tailored approaches to integrating telemedicine into healthcare systems that consider clinical effectiveness and patient experience.

Our study has several limitations. First, although the regression models adjusted for age, sex, and semester, the routinely collected dataset did not contain measures of comorbidity, illness severity, or socioeconomic status. Telephone users were substantially older (median 48 versus 31 years) and therefore likely to carry greater multimorbidity and clinical complexity; if, as is plausible, they were also more socioeconomically disadvantaged, residual confounding by indication would bias the comparison against telephone and inflate the apparent advantage of video. Standard adjustment for age and sex cannot resolve this imbalance, and the associations reported here should not be interpreted as causal evidence that video delivers higher-quality care; establishing causality would require designs that account for this selection, such as propensity-score, instrumental-variable, or randomised approaches. Second, the patient-reported and clinician-reported outcomes were derived from voluntary post-consultation prompts with substantial and modality-dependent non-response: the patient prompt was completed for 30.8% of video versus 13.4% of telephone consultations, and the clinician prompt for 34.5% versus 51.9%, respectively. The helpfulness, satisfaction, and in-person-need comparisons are therefore susceptible to selection and non-response bias and should be interpreted with caution; non-response was not equivalent across modalities. By contrast, recurrence was recorded automatically for all consultations and is not affected by this bias, although the questionnaire-based outcomes still rest on large samples (minimum *n* = 42,417). Third, the patient-reported outcomes were single-item, non-validated prompts used for routine quality monitoring (a binary helpfulness item and a 1–10 recommendation rating), and the clinician-reported need for in-person evaluation was likewise a single-item judgement; single-item measures lack the reliability and content coverage of validated multi-item instruments and are subject to measurement error and, for the clinician item, inter-clinician variability. Fourth, recurrence reflects intra-platform contacts only: we could not observe care sought from other providers, public primary care, or emergency departments, so total unresolved demand is likely underestimated. The absence of a recorded return does not necessarily indicate clinical resolution — it may reflect discontinuation of care, loss to follow-up, or care obtained elsewhere — and a recorded return does not necessarily indicate a failure of first-contact resolution (for example, planned follow-up for test results). Fifth, these findings derive from a single private, digital-first provider in Portugal; its population is likely younger, more digitally literate, and of higher socioeconomic status than the general primary care population, and differs from public systems such as the NHS or the Portuguese SNS, where digital access is more heterogeneous, so the findings are most applicable to comparable private, digital-first settings and extrapolation to public primary care requires caution. This analysis also focused on aggregate, cross-cutting quality indicators and did not stratify outcomes by clinical condition; condition-specific differences in resolution warrant dedicated investigation. Moreover, future studies evaluating telehealth services should consider using the Telehealth quality of care tool (TQoCT), recently developed by the World Health Organization [35].

## Conclusions

In conclusion, using the IOM framework as an organising lens, video consultations were associated with higher patient-reported helpfulness and lower short-term recurrence than telephone consultations, while telephone offered faster access and appeared adequate for routine and continuity tasks. These associations should not be interpreted causally: telephone users were substantially older, and unmeasured differences in comorbidity, illness severity, and socioeconomic status are likely to have confounded the comparison. The demographic skew — older patients concentrated in the telephone modality — nonetheless raises an equity concern about unequal access to information-richer consultations, rather than a demonstrated outcome disadvantage. Taken together, the findings support a more selective, clinically informed use of remote modalities and attention to digital equity, while underscoring that causal confirmation will require experimental or longitudinal designs. 

## Supplementary Information

Supplementary Material 1.

## Data Availability

The datasets used and/or analyzed during the current study are available from the corresponding author upon reasonable request.

## Abbreviations

CI: Confidence interval
EHRs: Electronic health records
IOM: Institute of Medicine (now National Academy of Medicine)
IQR: Interquartile range
ISO: International Organization for Standardization
NPS: Net Promoter Score
OR: Odds ratio
SMS: Short Message Service
STROBE: Strengthening the Reporting of Observational Studies in Epidemiology
TQoCT: Telehealth Quality of Care Tool
