# Supplementary material for: Comparing the quality of video and phone consultations in primary care telemedicine: a cross-sectional study of over 180,000 appointments
Source: BMC Prim Care. 2026 Jul 13;27:357. doi: 10.1186/s12875-026-03454-8 (PMC13651062; doi:10.1186/s12875-026-03454-8)
Supplement: Supplementary file 1 [file 12875_2026_3454_MOESM1_ESM.docx]

**Supplementary File S1 - Post-consultation Questionnaire (English translation)**

Manuscript: Comparing the Quality of Video and Phone Consultations in Primary Care Telemedicine: A Cross-sectional Study of Over 180,000 Appointments

Authors: Daniel Beirão et al.

Purpose: To reproduce the exact single-item prompts used to capture key quality indicators from patients and clinicians immediately after telemedicine encounters.

Notes:

- Original prompts were delivered in Portuguese within the telemedicine platform; this is the English translation for transparency.
- Items were optional; non-response accounts for missingness reported in the manuscript.
- The instrument was developed for this study as part of routine quality monitoring; no multi-item scales were used, and no psychometric validation was undertaken.
- Prompts were triggered automatically at the end of the consultation (patient view) and on case wrap-up (clinician view).

**Section A** — Patient prompts (displayed immediately after the consultation)

**A1**. “Was this telemedicine appointment helpful for your health concern?”

Response options: Yes / No

**A2**. “How likely are you to recommend this telemedicine service to a friend or family member?”

Response options: 1 to 10 (11-point numeric rating; 1 = Not at all likely, 10 = Extremely likely)

Note: Used to derive the Net Promoter Score (NPS).

**Section B** — Clinician prompt (displayed at the end of the consultation)

**B1**. “For this health concern, does the patient require an in-person evaluation?”

Response options: Yes / No
